# Treatment of a Neglected Patellar Tendon Rupture with a Modified Surgical Technique: Ipsilateral Semitendinosus Autograft Reconstruction with Suture Tape Augmentation

**DOI:** 10.1155/2018/2037638

**Published:** 2018-07-08

**Authors:** Sanjum P. Samagh, Fernando A. Huyke, Lucas Buchler, Michael A. Terry, Vehniah K. Tjong

**Affiliations:** Department of Orthopaedic Surgery, Northwestern University Feinberg School of Medicine, 259 East Erie Street, Suite 1350, Chicago, IL 60611, USA

## Abstract

Patellar tendon ruptures are rare, but debilitating injuries are typically seen in young active males in the third and fourth decades of life. They can occur as a single acute injury or from repetitive microtrauma weakening the tendon. Patients typically present complaining of knee pain, swelling, and an inability to perform a straight leg raise. Most conventionally, these injuries are classified as acute (less than two weeks) or chronic (greater than two weeks) based upon the timing of presentation. In patients with patellar tendon ruptures and inability to perform a straight leg raise, patellar tendon repair is most often recommended. A subset of patients with chronic patellar tendon ruptures, however, presents several months after their initial injuries. These neglected patella tendon ruptures present a particularly challenging clinical scenario in which primary repair is often difficult or not possible. This case report describes a modification to an existing surgical technique for reconstructing the patellar tendon using an ipsilateral semitendinosus tendon autograft with suture tape augmentation.

## 1. Introduction

Patellar tendon ruptures are rare, but debilitating injuries are typically seen in young active males in the third and fourth decades of life [1]. In older patient populations, these injuries are commonly the result of isolated trauma with a forceful indirect contraction of the quadriceps. In patients under 40, there is often underlying microtrauma from repetitive injury. A force of 17.5 times body weight is estimated to cause a rupture; by comparison, the patellar tendon experiences a force of 3.2 times body weight while ascending stairs. Forces on the patella are greatest at 60 degrees of flexion, and ruptures are most common at the distal pole of the patella as the tensile load is greatest at the insertion site [2].

Various classification systems have been used to describe patellar tendon ruptures. Siwek and Rao divided these injuries into acute (diagnosis and treatment less than two weeks after injury) or chronic (more than two weeks) [1]. Jobe's group later described patellar tendon injuries as transverse, *Z*-type, or inverted-U based on the tear configuration [3]. Hsu et al. further divided ruptures as distal pole, tendon midsubstance, or tibial tubercle [4]. Currently, the Siwek and Rao classification of patellar tendon ruptures as acute or chronic and the surgical technique for treatment of ruptures seem to be the main perioperative factors that determine patient outcomes postoperatively [5–7].

Neglected patellar tendon ruptures are challenging to manage secondary to the retraction of the patella proximally and scarring of the surrounding tissues. Several surgical techniques have been described for treatment of chronic ruptures, but the ideal approach remains debatable [8, 9]. While studies have demonstrated less favorable outcomes in chronic patellar tendon ruptures compared to acute injuries, augmentation strengthens the construct and allows earlier return to range of motion [10–12]. Options for augmentation include autologous semitendinosus and gracilis tendon grafts, contralateral bone-patellar tendon-bone autografts, turndown of the quadriceps tendon, Achilles and extensor mechanism allografts, and artificial materials [13–19]. Recent publications advocate for the use of autologous ipsilateral hamstring tendon graft reconstruction as a surgical option for the management of chronic patellar tendon rupture due to its association with good patient functional recovery and return to preinjury levels of activity [20–22]. The purpose of this case report is to share a modification of this surgical technique for reconstructing a neglected patellar tendon rupture: an autologous ipsilateral hamstring tendon graft reconstruction with additional suture tape augmentation.

## 2. Patient Presentation and Physical Examination

In delayed presentations of patellar tendon rupture, a patient may complain of difficulty with activities requiring active knee extension such as stair climbing or rising from a seated position. On physical exam, some active extension may be possible, but this is typically weak, and an extensor lag may be present. The patient sometimes presents with no effusion or hemarthrosis of the knee, but some mild swelling is possible. A palpable gap in the tendon may or may not be present as a scar often fills in over time [23].

Plain radiographs of the knee in the lateral plane and comparing this to the contralateral leg often demonstrate patella alta with an Insall-Salvati ratio greater than 1.2 [24]. Ultrasound or an MRI may be useful in qualifying the amount of tendon degeneration and other associated knee injuries in delayed diagnoses.

The patient in this case was a 25-year-old obese male with a past medical history of borderline hypertension and diabetes well controlled with medication and a moderate level of physical activity that heard a pop in his knee after playing basketball. The patient ignored his knee injury until 5 months later when he presented to a clinic with instability in his knee, inability to fully straighten his leg, and anterior knee pain with flexion. On physical exam, the patient's preoperative range of motion was 10 to 100 degrees. The patient began experiencing anterior knee pain and tightness after 90 degrees of flexion.

## 3. Surgical Technique

### 3.1. Patient Positioning

The patient is brought to the operative suite and positioned supine on the operating room table, and general anesthesia is induced. Prior to draping, the contralateral knee is placed in 60 degrees of flexion, and the length of the native patellar tendon is measured (4.5 cm in the case pictured). The operative extremity is prepped and draped to the proximal thigh. A sterile triangle is placed to hold the knee in 60 degrees of flexion. The distance between the inferior pole of the patella and the tibial tubercle is then measured on the injured extremity (8.0 cm). A sterile tourniquet is applied to the thigh but will only be inflated if needed.

### 3.2. Surgical Approach and Repair

**Figure 1:**
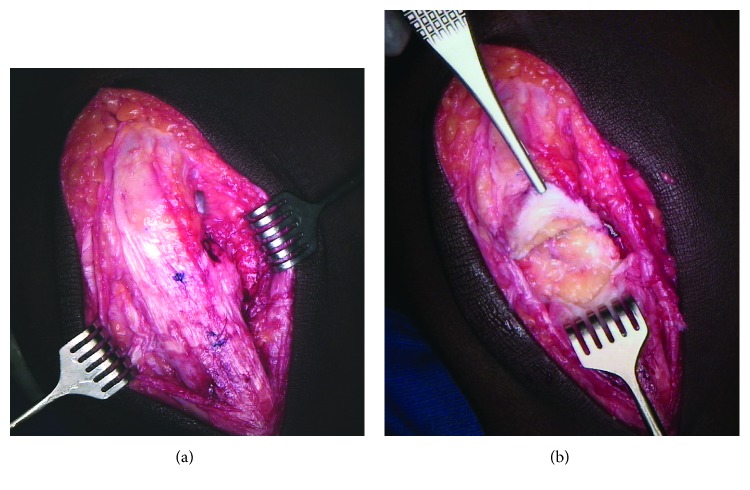
(a) The peritenon is identified superiorly, and peritendonous flaps are elevated to reveal the chronic, scarred patellar tendon rupture (approximately 3.5 cm in the midsubstance of the tendon). (b) The chronically scarred portion of the tendon is then debrided along with the medial and lateral retinacula. A Cobb elevator is used to release the superficial and deep aspects of the quadriceps tendon from the surrounding tissues.

A midline longitudinal incision is made from the superior pole of the patella to the tibial tubercle through the skin and subcutaneous tissue. The underlying fascia is identified and incised longitudinally. The peritenon is identified superiorly, and the peritendonous flaps are elevated to reveal the chronic, scarred patellar tendon rupture (approximately 3.5 cm in the midsubstance of the tendon) (Figure 1(a)). The scarred portion of the tendon is debrided along with any redundancy of the medial and lateral retinacula. A Cobb elevator is used to release the superficial and deep aspects of the quadriceps tendon from the surrounding tissues (Figure 1(b)).

**Figure 2:**
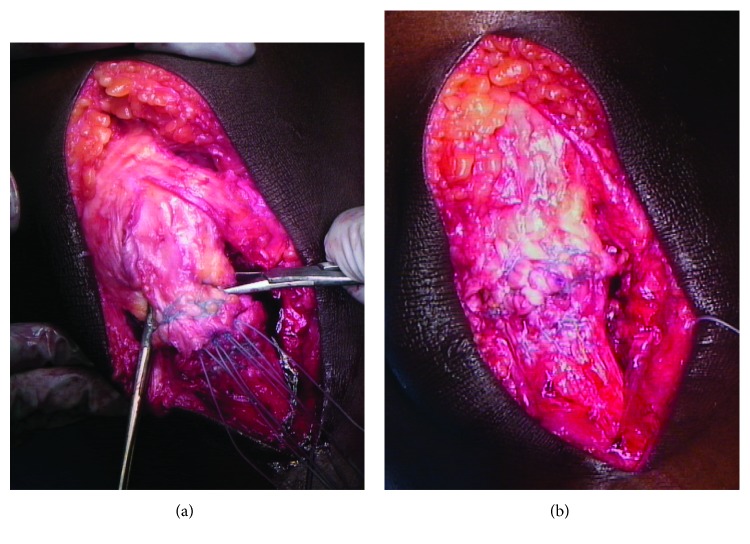
(a) A four-stranded end-to-end repair is then undertaken using a #5 FiberWire (Arthrex, Naples, FL) in a Krackow fashion. (b) To secure the repair, a #2 FiberWire (Arthrex, Naples, FL) is used in a running-locking fashion to imbricate the elongated medial and lateral retinacula and oversew the tendon repair.

The vastus medialis is left intact, and the surgeon ensures that the patella can be reduced to its native position at 60 degrees of flexion as measured on the contralateral knee preoperatively. A four-stranded end-to-end repair is then undertaken using a #5 FiberWire (Arthrex, Naples, FL) in a Krackow fashion (Figure 2(a)). A #2 FiberWire (Arthrex, Naples, FL) is used in a running-locking fashion to imbricate the elongated medial and lateral retinacula and oversew the tendon repair (Figure 2(b)).

Next, the semitendinosus autograft is harvested. The insertion of the pes anserine is visualized through the same incision, and the semitendinosus tendon is identified and detached from its insertion. Once rid of adhesions, the tendon is harvested in standard fashion with a closed tendon harvester. The graft is prepared on the back table with both ends tied in a Krackow manner using #2 FiberWire sutures and then sized when doubled (6.5 mm).

**Figure 3:**
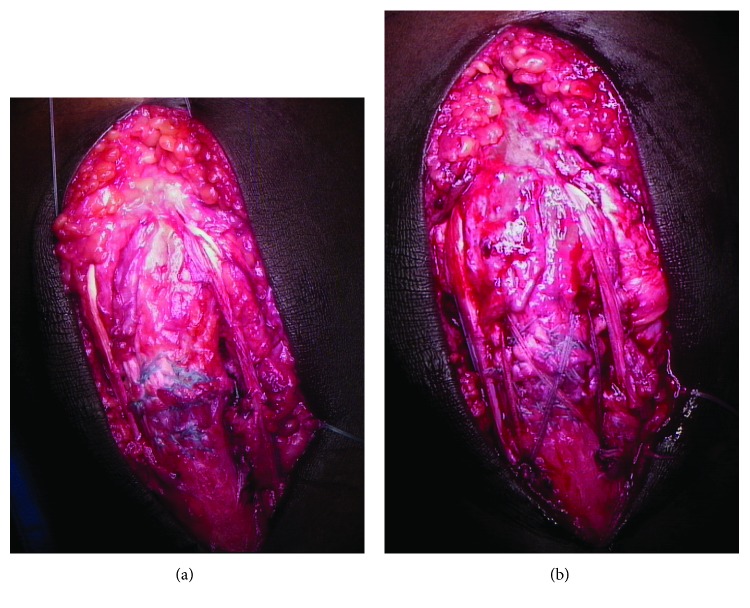
(a) The semitendinosus autograft is passed through the bone tunnel from medial to lateral and courses along the lateral aspect of the native patellar tendon. Small rents are created in the medial and lateral retinacula at the level of the superior pole of the patella, and the graft is woven transversely through the distal quadriceps tendon from lateral to medial. The graft is then brought down along the medial border of the native patellar tendon and passed through the bone tunnel from lateral to medial. The free ends cross in opposite directions within the tibial bone tunnel and are secured within the tibial tunnel using two interference screws (Tenodesis Screw, BioComposite, 6.25 × 15 mm; Arthrex, Naples, FL)—one from medial to lateral and one from lateral to medial. (b) An InternalBrace (Arthrex, Naples, FL) is used to augment and protect the reconstruction. Two 4.75 × 15 mm SwiveLock BioComposite suture anchors (Arthrex, Naples, FL) are placed into the distal pole of the patella securing the midpoint of one 2 mm FiberTape suture tape (Arthrex, Naples, FL) each. The free limbs of the FiberTape (Arthrex, Naples, FL) cross the repair site—one limb straight inferior and the other in an “X” fashion—and are then secured to the tibial tubercle using two 4.75 × 15 mm SwiveLock BioComposite suture anchors (Arthrex, Naples, FL).

A tibial bone tunnel is created using an appropriately sized (6.5 mm) cannulated reamer (Arthrex, Naples, FL), 3 cm distal to the tibial tubercle and 2 cm posterior from the anterior tibial crest (6.5 × 40 mm). The graft is passed through the bone tunnel from medial to lateral and courses along the lateral aspect of the native patellar tendon. Small rents are created in the medial and lateral retinacula at the level of the superior pole of the patella, and the graft is woven transversely through the distal quadriceps tendon from lateral to medial. The graft is then brought down along the medial border of the native patellar tendon and passed through the bone tunnel from lateral to medial. The free ends cross in opposite directions within the tibial bone tunnel and are secured using interference screws (Tenodesis Screw, BioComposite, 6.25 × 15 mm; Arthrex, Naples, FL)—one from medial to lateral and one from lateral to medial (Figure 3(a)).

Finally, an InternalBrace (Arthrex, Naples, FL) is used to augment and protect the reconstruction. Two 4.75 × 15 mm SwiveLock BioComposite suture anchors (Arthrex, Naples, FL) are placed into the distal pole of the patella securing the midpoint of one 2 mm FiberTape suture tape (Arthrex, Naples, FL) each. The free limbs of the FiberTape cross the repair site—one limb straight inferior and the other in an “X” fashion—and are then secured to the tibial tubercle using two 4.75 × 15 mm SwiveLock BioComposite suture anchors (Figure 3(b)). The tension of the InternalBrace is set such that the knee can be passively flexed to 90 degrees. The wounds are thoroughly irrigated, closed, and dressed in the surgeon's usual sterile fashion.

### 3.3. Postoperative Rehabilitation

The patient was placed in a locked hinged knee brace and instructed to bear weight as tolerated with crutches in extension for 6 weeks. A supervised physical therapy program lasting 4 months was recommended. At 6 weeks, the patient was able to perform passive and active flexion to 90 degrees. At 12 weeks, the patient discontinued the brace and began zero-resistance straight leg raise exercises, as recommended. The patient began quadriceps strengthening at 12 weeks and ultimately should return to impact cardiovascular activity after 20–24 weeks. This current patient has had a six-month follow-up. He has active range of motion from 0–120 degrees with no extensor lag and has returned to sporting activity.

## 4. Discussion

This report details our technique for treatment of neglected patellar tendon ruptures with patellar tendon reconstruction using a semitendinosus tendon autograft and suture tape augmentation. While the vast majority of patellar tendon ruptures are diagnosed and treated in a relatively timely manner, some go undiagnosed and lead to chronic injuries with significant retraction and scarring of the tendon. Although the prevalence and incidence of these undiagnosed injuries are unknown, their surgical management is challenging due to delay of diagnosis. Nonoperative management has limited indications and does not restore the function of the effected extremity. Late repair of chronic patellar tendon ruptures was first described in 1927, and several techniques have been described [25]. We advocate for autograft reconstruction as a method of tendon augmentation including the passage and incorporation through bone tunnels. Allograft reconstruction has an increased risk of infection and weakness secondary to radiation of the tendon graft. In addition, animal models have demonstrated that allograft extensor mechanism reconstruction shows a reduction in vascularity and cellularity when histologically compared to autograft reconstruction [26]. We also elected to use nonabsorbable sutures because we believe they add strength and longevity to the repair, which have been seen in a literature review of acute patellar tendon repair and an Achilles tendon repair model [27, 28].

The best method of treatment for neglected patellar tendon ruptures is controversial; however, we believe the technique above results in a robust construct that allows for the return of strength and function of the knee through early rehabilitation. Given the rare occurrence of neglected patellar tendon ruptures, there is a paucity of comparative literature to guide management. Future long-term studies are necessary to assess long-term outcomes with this injury and surgical technique.

## 5. Conclusion

Chronic patellar tendon rupture is a rare injury, and consensus for a gold standard approach to surgical management has yet to be reached. Previous cases of ipsilateral semitendinosus autograft reconstruction have shown positive outcomes and relatively rapid return to physical activity with rehabilitation. Our modified surgical technique involving suture tape augmentation provides a potentially stronger construct in addition to providing excellent range of motion and return to sporting activity at a six-month follow-up.
